# Efficacy of pre-exposure prophylaxis to prevent SARS-CoV-2 infection after lung transplantation: a two center cohort study during the omicron era

**DOI:** 10.1007/s15010-023-02018-7

**Published:** 2023-03-16

**Authors:** Jens Gottlieb, Susanne Simon, Jürgen Barton, Michaela Barnikel, Marcus Bachmann, Merle-Sophie Klingenberg, Tobias Veit, Nikolaus Kneidinger

**Affiliations:** 1 Department of Respiratory Medicine, Hannover Medical School, Hannover, Germany; 2 German Center for Lung Research (DZL), Hannover, Germany; 3 Department of Medicine V, Comprehensive Pneumology Center (CPC-M), German Center for Lung Research (DZL), University Hospital, LMU Munich, Marchioninistrasse 15, 81377 Munich, Germany

**Keywords:** Lung transplantation, COVID-19, Antibodies, Monoclonal, SARS-CoV-2, Prophylaxis, Tixagevimab, Cilgavimab, Pre-exposure prophylaxis

## Abstract

**Purpose:**

Lung transplant (LTx) recipients are at risk for poor outcomes from coronavirus disease 2019 (COVID-19). The aim of the study was to assess the outcome of patients receiving pre-exposure prophylaxis (PrEP) with tixagevimab and cilgavimab after LTx.

**Methods:**

All LTx recipients with outpatient visits from February 28th to October 31st, 2022 at two German centers were included. Baseline characteristics were recorded and patients followed until November 30rd, 2022. Infections with SARS-CoV-2, disease severity, and COVID-19-associated death were compared between patients with and without PrEP.

**Results:**

In total, 1438 patients were included in the analysis, and 419 (29%) received PrEP. Patients receiving PrEP were older and earlier after transplantation, had lower glomerular filtration rates, and lower levels of SARS-CoV-2-S antibodies. In total, 535 patients (37%) developed SARS-CoV-2 infection during a follow-up of median of 209 days. Fewer infections occurred in patients with PrEP during the study period (31% vs. 40%, *p* = 0.004). Breakthrough SARS-CoV-2 infections after PrEP occurred in 77 patients (19%). In total, 37 infections (8%) were severe or critical. No difference in severity of COVID-19 was observed between patients with and without PrEP. There were 15 COVID-19-associated deaths (*n* = 1 after PrEP). Compared to matched controls, there was a non-significant difference towards a lower risk for moderate to critical COVID-19 (*p* 0.184).

**Conclusion:**

The number of SARS-CoV-2 infections was lower in LTx recipients with PrEP. Despite being at higher risk for worse outcome severity of COVID-19 and associated mortality were similar in patients with and without PrEP.

**Supplementary Information:**

The online version contains supplementary material available at 10.1007/s15010-023-02018-7.

## Introduction

Transplant recipients are at risk for poor outcomes from coronavirus disease 2019 (COVID-19) due to frequent medical comorbidities and the presence of immunosuppression. In a US retrospective analysis in the pre-delta era of the pandemic 78% of infected transplant recipients were hospitalized and 19% died within 28 days [1]. Observational cohort studies suggest that lung transplantation recipients with COVID-19 have higher mortality in comparison to other solid organ transplant recipients [2].

Over the course of the pandemic a variety of antiviral measures have been developed or repurposed which resulted in a decline in both hospitalization and mortality rates [3, 4]. The main recommended strategy for preventing COVID-19 infection is vaccination. Vaccination is the most effective way to protect against severe COVID-19 and is highly recommended for all patients at risk including transplant patients. However, poor vaccination response despite several attempts has been consistently reported in solid organ transplant recipients [5, 6].

Monoclonal antibodies, which bind and neutralize the severe acute respiratory syndrome coronavirus type 2 (SARS-CoV-2) in infected individuals have been proposed as pre-exposure prophylaxis (PrEP) to prevent symptomatic COVID-19 [7–10]. Tixagevimab and cilgavimab are two SARS-CoV-2–neutralizing monoclonal antibodies that are derived from antibodies obtained from SARS-CoV-2 infected persons and modified for half-life extension. The combination has been shown to neutralize SARS-CoV-2 in vitro. In a randomized controlled trial with 5197 participants, during the pre-delta pandemic era, a single intramuscular dose (150 mg each) of tixagevimab and cilgavimab as PreEP reduced the incidence of symptomatic SARS-CoV-2 infections after 6 months from 1.8% in placebo to 0.3% [10]. However, the randomized trial was performed during the pre-omicron era of the pandemic when the incidence of COVID-19 was significant lower. Further, only a minority of participants were immunosuppressed [10]. Some retrospective studies on mainly kidney transplant recipients indicate a lower risk of breakthrough infections after PrEP than vaccination only [11–14].

The aims of this study were to assess the outcome of patients receiving PrEP after lung transplantation, and its efficacy during the omicron wave.

## Methods

A retrospective analysis in the two largest German lung transplant centers (Hannover and Munich) was performed. All adult patients attending the specialised lung transplant outpatient follow-up clinics during between February 28th 2022 and October 31st 2022 were included. Follow-up was recorded until November 30th 2022 or until death whichever occurred first.

The study was performed according to the declaration of Helsinki of 1975. The study was approved by the central institutional ethics committee (Munich, Germany; project number 22-0894).

Number and dates of previous vaccinations, type of vaccine, previous infections with SARS-CoV-2 and last available antibodies against Spike-protein (SARS-CoV-2-S) were recorded. Antibody status was classified in unknown, negative (binding antibody units (BAU) < 50/ml), low (BAU 50–250/ml), and positive (BAU > 250/ml). Patients after PrEP were tested for antibodies against Spike-protein (SARS-CoV-2-S) during follow-up on each visit.

Pre-exposure prophylaxis was offered to patients with an inadequate humoral immune response (< 250 BAU /ml) after full vaccination or on a case-based decision by the treating physician. Contraindications were body weight below 40 kg or bleeding disorders including the inability for temporal interruption of anticoagulation.

Tixagevimab-cilgavimab as PrEP was administered each as bilateral 150 mg (single dose) or 300 mg (double dose) intramuscular intragluteal injections. Double dosing of PrEP was preferred in both centers according to national recommendations [15] but the hospital pharmacy dispensed single-dosed tixagevimab–cilgavimab occasionally following the manufacturer´s specification.

Adverse events were not routinely collected but patients were encouraged to report them by telephone and side effects were asked about on the next scheduled visit.

Baseline characteristics (age, sex, underlying disease, lung transplant procedure: bilateral, unilateral or combined organ transplantation and immunosuppressive regimen) and comorbidities (diabetes mellitus, obesity, chronic lung allograft dysfunction, and estimated glomerular filtration rate (GFR)) were recorded. Chronic lung allograft dysfunction (CLAD) was defined according to recently established criteria [16].

SARS-CoV-2 infections were defined as any positive polymerase chain reaction (PCR) test. SARS-CoV-2 infections were recorded during the study period and follow-up after infection was performed for at least 28 days. COVID-19 severity was adjudicated according to the modified WHO scale [17] with a recording of the highest stage during follow-up after infection. In brief, a mild disease was defined as constitutional symptoms without signs of pneumonia or respiratory failure. A moderate disease had signs of pneumonia without respiratory failure (blood oxygen saturation (SpO_2_) ≥ 92%, no use of oxygen). Patients admitted with non-severe disease, e.g., for non-pulmonary reasons within 28 days after infection were graded as a moderate disease. Severe disease was defined as respiratory rate ≥ 30/min, SpO_2_ < 92%, and use of oxygen or opacities > 50% on pulmonary imaging. A critical disease was defined as respiratory failure with the need of mechanical ventilation support, the presence of septic shock, or multiple organ failure. COVID-19-associated death was defined as death during hospitalization with at least severe COVID-19.

Dates of symptom onset, and SARS-CoV-2 infection proven by PCR and applied treatments, respectively, were recorded. In the case of asymptomatic patients, the date of positive SARS-CoV-2 PCR was regarded as the date of disease onset.

Patients after lung transplantation were routinely treated with a triple-drug immunosuppressive regimen containing calcineurin inhibitor, prednisolone and an antimetabolite (mycophenolate, azathioprine) or proliferation signal inhibitor. In lung transplant recipients with SARS-CoV-2 infection antimetabolites were temporarily reduced or interrupted. Specific antivirals for early treatment during the study period were tixagevimab–cilgavimab, sotrovimab, remdesivir, molnupiravir and nirmatrelvir–ritonavir. Treatment options used late in the course of the disease like dexamethasone and tocilizumab were recorded but not analyzed.

### Statistics

Metric variables were expressed as medians and 25 and 75% percentile. Univariate analyses were performed using the Mann–Whitney test for continuous variables and chi-square test for categorical variables. Patient subgroups with and without PrEP were matched for age, sex, vaccination status, GFR (± 5 ml/min/1.73 m^2^), and age (± 5 years). Within the matched population, a survival analysis was performed using the Kaplan–Meier method for the differences in survival until any SARS-CoV-2 infection or until at least moderate SARS-CoV-2 infection. Survival differences between groups were compared using the log-rank test.

Data were analyzed as observed without imputation of missing values.

## Results

**Fig. 1:**
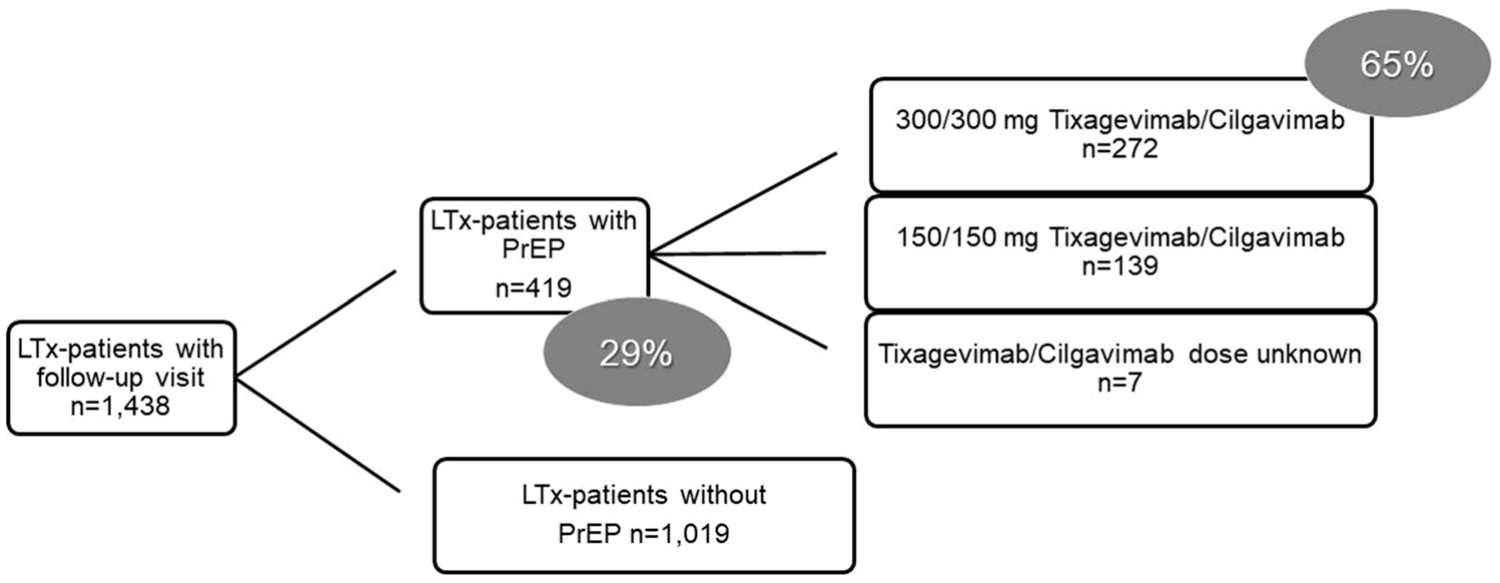
Flowchart of patients. *LTx* lung transplantation, *PrEP* preexposure prophylaxis

In total, 1,438 lung transplant recipients had at least one visit during the study period and were included in the analysis (Fig. 1). Thereof, 419 patients (29%) received PrEP and 272 patients (65%) received a double dose of tixagevimab–cilgavimab. Re-dosing of tixagevimab–cilgavimab after 6 months was performed in 23 patients (5.5%). Patient characteristics are displayed in Table 1.

**Table 1:** Patient demographics

	Lung transplant recipients in follow-up, total *n* = 1438	Lung transplant recipients with PrEP in follow-up *n* = 419	Lung transplant recipients without PrEP in follow-up *n* = 1019	*p* value^*^
Sex, *n* (%)				
Male	773 (54)	220 (53)	553 (54)	0.584
Female	665 (46)	198 (48)	467 (46)	
Age at visit, median years (25, 75% percentile)	58 (48, 64)	60 (52, 65)	57 (46, 64)	< 0.001
Time after transplant, median years (25, 75% percentile)	5.4 (2.6, 9.4)	4.3 (2.4, 8.5)	5.8 (2.8, 9.8)	0.006
Lung transplant procedure, *n* (%)				
Bilateral	1328 (93)	383 (92)	945 (93)	0.004
Unilateral	72 (5)	30 (7)	42 (4)	
Combined	35 (2)	4 (1)	31 (3)	
Underlying disease, *n* (%)				
Emphysema	377 (26)	111 (27)	266 (26)	< 0.001
Pulmonary vascular disease	100 (7)	13 (3)	87 (9)	
Cystic fibrosis/bronchiectasis	315 (22)	81 (19)	234 (23)	
Fibrosis/interstitial lung disease	535 (37)	183 (44)	352 (35)	
Other	108 (8)	29 (7)	79 (8)	
Preexisting CLAD, *n* (%)	473 (33)	117 (28)	356 (35)	
Immunosuppression, *n* (%)				
Tacrolimus	1139 (79)	356 (85)	783 (77)	< 0.001
Ciclosporine	299 (21)	62 (15)	237 (23)	
Mycophenolate	1328 (92)	378 (90)	950 (95)	0.001
Azathioprine	31 (2)	10 (2)	21 (2)	
Proliferation signal inhibitor	101 (7)	46 (11)	55 (5)	
Dual Immunosuppression	50 (4)	24 (6)	26 (3)	0.002
Comorbidities, *n* (%)				
GFR ml/min/1.73 m^2^, median (25, 75% percentile)	50 (35, 68)	46 (35, 61)	52 (36, 70)	< 0.001
Body mass index, kg/m^2^ (25, 75% percentile)	22 (19, 24)	22 (19, 24)	22 (19, 24)	0.071
Pre-study SARS-CoV2 infection, *n*/*n* (%)	115 (11)	5 (3)	110 (15)	
Vaccination status, *n* (%)				
Missing data	48 (3)	15 (4)	33 (3)	
No or incomplete vaccination	133 (9)	48 (11)	85 (8)	0.058
Full vaccination (at least 3 doses)	1.257 (87)	355 (85)	902 (89)	
Humoral immune response after vaccine, *n* (%)				
Missing data	659 (46)	25 (6)	626 (63)	
< 50 BAU /ml	423 (29)	333 (80)	173 (17)	
50–250 BAU /ml	167 (12)	33 (8)	108 (11)	< 0.001
> 250 BAU/ml	189 (13)	28 (7)	112 (11)	
Follow-up, median days (25, 75% percentile) after first visit during study period	203 (144, 245)	211 (154, 251)	198 (140, 246)	
				0.016

*BAU* binding antibody units, *CLAD* chronic lung allograft dysfunction, *GFR* glomerular filtration rate, *PrEP* pre exposure prophylaxis

Patients receiving PrEP were older, had lower GFR, and were earlier after transplantation. Furthermore, despite a high vaccination efforts patients with PrEP had more often a negative antibody response, taking a high number of missing values in patients without PrEP into account (Table 1).

A total of 535 patients (37%) developed a SARS-CoV-2 infection during the study period. Fewer infections occurred in patients with PrEP during the study period (31% vs 40%, *p* = 0.004). Breakthrough SARS-CoV-2 infections occurred in 77 patients (19%) in a linear fashion after PrEP (supplemental figure). Follow-up after PrEP was a median of 209 (99, 217) days.

Patients with PrEP received less frequently early antiviral therapy for COVID-19 compared to patients without PrEP (34% vs. 25%, *p* = 0.315) without being statistically different. In the case of early antiviral therapy, patients with PrEP received more often molnupiravir (27% vs. 14%, *p* = 0.01) and less frequently remdesivir (18% vs. 28%, *p* = 0.11) compared to patients without PrEP.

Most infections (*n* = 413, 77%) within the study period were asymptomatic or mild without differences between the groups. Furthermore, the proportion of patients with moderate, severe or critical disease were not different between groups (Table 2).

**Table 2:** Patient outcomes

	Lung transplant recipients with PrEP in follow-up *n* = 419	Lung transplant recipients without PrEP in follow-up *n* = 1019	*p* value
SARS-CoV2 infection during study period, *n* (%)	128 (31)	407 (40)	0.004
SARS-CoV2 infection after Evusheld, *n* (%)	77 (19)	–	
COVID-19 severity* during study period, *n* (%)			
Asymptomatic	2 (3)	22 (6)	0.760
Mild	65 (84)	324 (80)	
Moderate	5 (5)	28 (7)	
Severe	2 (3)	16 (4)	
Critical	3 (5)	16 (4)	
Early treatment of SARS-CoV2-Infection*, *n* (%)			
Missing data	1 (1)	24 (6)	0.001
None/reduction of immunosuppression	26 (34)	109 (25)	
Remdesivir	14 (18)	115 (28)	
Molnupiravir	21 (27)	57 (14)	
Sotrovimab	3 (4)	86 (21)	
Tixagevimab/Cilgavimab	5 (6)	14 (3)	
Death*, *n* (%)	5 (1)	29 (3)	0.032
COVID-19 associated death*, *n* (%)	1 (0)	14 (1)	0.500

*PrEP* pre-exposure prophylaxis

*Infections after Evusheld only in patients with PrEP

In total, 34 patients (2.4%) died during the study period with no difference in COVID-19-associated deaths between groups (*n* = 15, 2.8% of those infected) as shown in Table 2.

To reduce imbalances between the groups patients with PrEP were matched to patients without PrEP (Table 3). Differences in time after transplant persisted in matched groups with patients receiving PrEP being earlier after transplant at their first visit during the study period. Furthermore, difference in early antiviral therapies remained. Significantly more patients with PrEP (44% vs. 10%) received no specific antiviral therapy after developing SARS-CoV-2 infection. Due to the high number of missing SARS-CoV-2-S values patients could not be matched according to antibodies levels.

**Table 3:** Matched Population

	Lung transplant recipients with PrEP in follow-up *n* = 373	Lung transplant recipients without PrEP in follow-up *n* = 373	*p* value
Sex, *n* (%)			
Male	196 (52)	196 (52)	1.00
Female	177 (48)	177 (48)	
Age at visit, median years (25, 75% percentile)	60 (53, 65)	60 (53, 64)	0.815
Time after transplant, median years (25, 75% percentile)	4.4 (2.4, 8.5)	6.3 (3.6, 10.2)	< 0.001
Comorbidities, *n* (%)			
GFR ml/min/1.73m^2^, median ((25, 75% percentile)	46 (34, 61)	48 (34, 61)	0.718
Body mass index, kg/m^2^ (25, 75% percentile)	22 (19, 25)	21 (19, 24)	0.008
Pre-study SARS-CoV2 infection, *n*/*n* (%)	5 (3)	44 (13)	< 0.001
Vaccination status, *n* (%)			
No full vaccination	27 (7)	27 (7)	1.00
Full vaccination (3 + doses)	341 (92)	341 (92)	
Follow-up, median days (25, 75% percentile)^#^	146 (81, 203)	147 (88, 207)	0.394
SARS-CoV2 infection during study period, n (%)	120 (32)	148 (40)	0.058
SARS-CoV2 infection after Evusheld, *n* (%)	72 (19)	–	
COVID-19 severity* during study period, *n* (%)			
Asymptomatic	2 (3)	8 (6)	0.461
Mild	56 (84)	123 (85)	
Moderate	3 (5)	9 (6)	
Severe	2 (3)	2 (1)	
Critical	4 (3)	3 (2)	
Early treatment of SARS-CoV2-Infection*, *n* (%)			
Missing data	1 (1)	15 (10)	< 0.001
None/reduction of immunosuppression	32 (44)	45 (30)	
Remdesivir	14 (19)	71 (48)	
Molnupiravir	17 (24)	20 (14)	
Sotrovimab	3 (4)	19 (13)	
Tixagevimab/Cilgavimab	5 (6)	0 (0)	
Death*, *n* (%)	3 (1)	10 (3)	0.009
COVID-19 associated death*, *n* (%)	1 (0)	4 (1)	0.373

*BAU* binding antibody units, *CLAD* chronic lung allograft dysfunction, *GFR* glomerular filtration rate, *PrEP* pre exposure prophylaxis

*Infections after Evusheld only in patients with PrEP

**Fig. 2:**
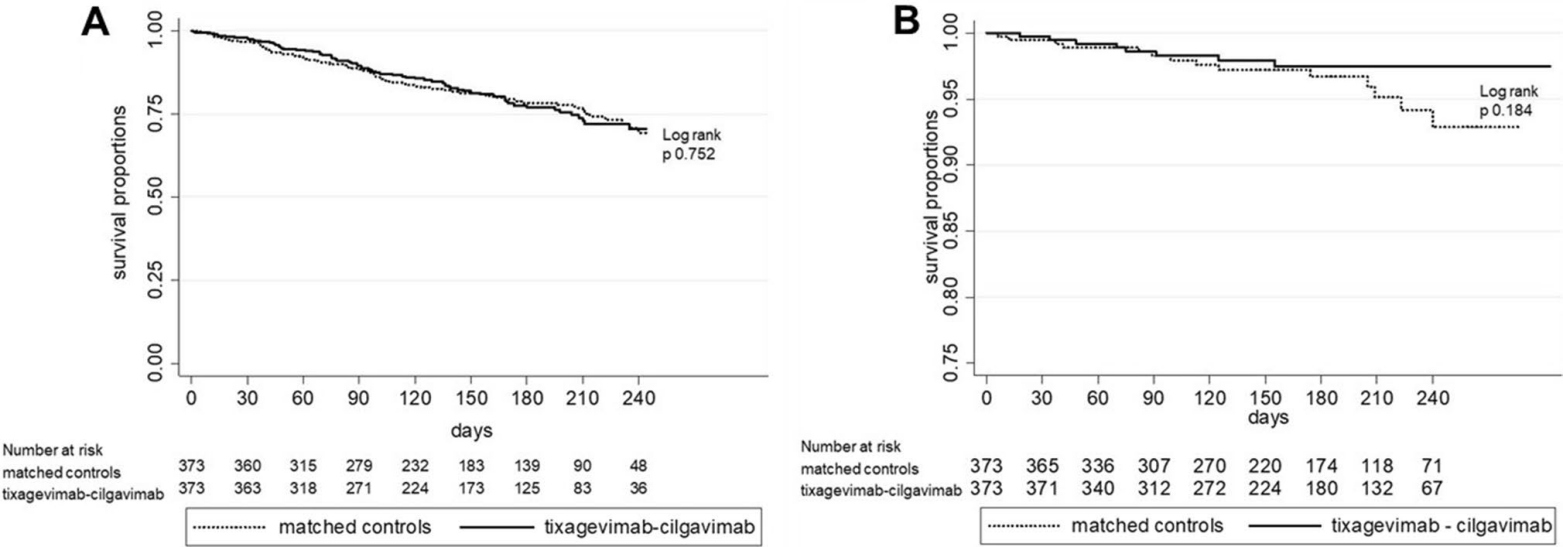
SARS-CoV-2 IgG- anti-spike protein antibodies after pre-exposure prophylaxis. *BAU* binding antibody units, trendline displays linear regression

No difference in COVID-related deaths was noted between matched groups. Survival without SARS-CoV-2 infection was not different after PrEP compared to matched controls (Fig. 2A). A non-significant difference towards better survival until moderate-to-critical disease was noted after PrEP compared to matched controls (Fig. 2B).

**Fig. 3:**
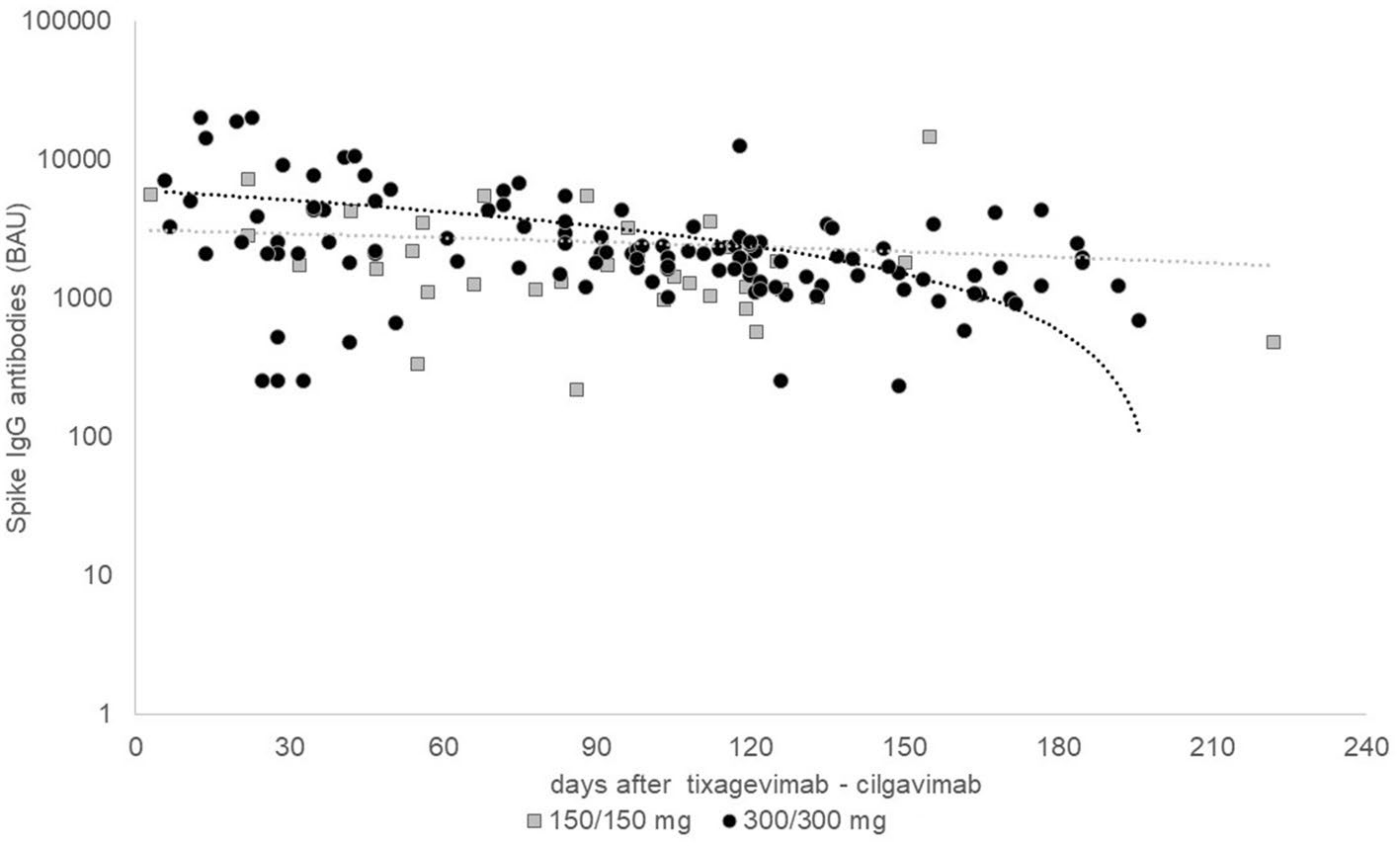
Kaplan–Meier survival curves in the matched population until any SARS-CoV-2 infection (panel **A**) and survival until moderate to critical SARS-CoV-2 infection (panel **B**)

In 114 lung transplant recipients (27%) after PrEP SARS-CoV-2-S antibody measurements were available. Thereof, eight participants had multiple measurements. Measurements were above 1.000 BAU/ml in 88% and 83% after receiving a double and single dose PrEP, respectively. SARS-CoV-2-S antibody levels were only slightly affected by the time since administration as shown in Fig. 3.

Patient-reported side effects were limited to injection site reactions. A single patient reported a rash 7 days after injection, which rapidly responded to topical treatment. No systemic reactions or hospitalizations associated with PrEP were reported.

## Discussion

Breakthrough SARS-CoV-2 infections occurred frequently in lung transplant recipients. The number of SARS-CoV-2 infections was lower in lung transplant recipients with PrEP compared to recipients without PrEP. Lung transplant recipients with PrEP were older, had a worse kidney function, were earlier after transplantation and had lower levels of SARS-CoV-2-S antibodies, indicating a selection bias for individuals at risk for worse outcome. Furthermore, in the case of COVID-19 patients with PrEP received less frequent antiviral therapies. Despite these differences, the course of COVID-19 and associated outcome were similar between patients with and without PrEP. Analysis attempting to reduce imbalance between patients revealed similar outcome with a non-significant difference toward less severe COVID-19 after PrEP compared to matched controls.

Our study demonstrates the high number of lung transplant recipients infected by SARS-CoV-2 during the omicron era dominated by the sublineages BA.2 and BA.5. Despite the increased number of affected individuals the severity of the disease has declined in lung transplant recipients, which is in line with previous reports on non-transplant individuals [4]. COVID-19-associated mortality has been reported of up to 50% in early single-center studies [18, 19]. The proportion of COVID-19-associated death rate declined from 28 to 17%, 6%, and 1.3% in the pre-delta, delta, omicron BA.1 and BA.2 and BA.5 era (this study), respectively [4, 20]. Despite improved outcome there is a need for additional antiviral measures to further reduced COVID-19-associated morbidity and associated long-term sequaele. In particular, since antibody response after vaccination is poor and the efficacy of early antiviral therapies is debated in transplant recipients [20–22].

In this respect, the monoclonal antibody combination of tixagevimab and cilgavimab was approved for the PrEP of COVID-19 and widely recommended for moderately and severely immunocompromised patients. In our study, approximately one-third of lung transplant recipients received PrEP. PrEP was offered to patients with inadequate humoral immune response after full vaccination or on a case based decision by the treating physician. This likely resulted in a selection for lung transplant recipients with a higher risk for worse outcome. Patients who received PrEP were older, had a lower GFR, were earlier after transplantation and had lower levels of SARS-CoV-2-S antibodies. In this line age and a low GFR were the only risk factors for a severe or critical COVID-19 in a previous multicenter study of lung transplant recipients with COVID-19 during the early Omicron wave [20]. The time after transplantation has not been linked to the outcome of COVID-19 but may indicate a selection for patients with a higher level of immunosuppression. Furthermore, the selection according presence or absence of a sufficient humoral response is likely the main bias when comparing lung transplant recipients with and without PrEP. Although being assumed to be at higher risk for worse outcome lung transplant recipients with PrEP received less frequent early antiviral therapy in the case of a SARS-CoV-2 infection. Reasons for this contradiction have not been explored but are possibly associated with favorable risk assessment of patients after having received PrEP compared to the risk assessment of patients without PrEP.

Despite an obvious selection for patients at risk for worse outcome and the fact that antiviral therapies were less frequently used, outcome of COVID-19 was similar between lung transplant recipients with PrEP and without PrEP. The missing difference in favor for PrEP might seem disappointing at first glance. However, keeping the imbalance between the groups in mind the non-inferiority of patients with PrEP may indicate a treatment benefit.

To exclude differences between the groups patient with PrEP were matched to respective controls, taking factors associated previously shown to be associated with worse outcome into account. Even though obvious differences for time after transplantation and SARS-CoV-2-S antibody levels remained. In matched patients, outcome remained similar between the groups with a non-significant difference in favor for lung transplant recipients with PrEP. With respect to the small outcome difference and all limitations of a matched-subject design results, however, have to be interpreted cautiously.

Further limited by the retrospective study design is the analysis of the number of SARS-CoV-2 infections between the groups. In a follow-up of approximately 200 days, the number of SARS-CoV-2 infections was lower in patients with PrEP. Despite a close follow-up and awareness of lung transplant recipients for infections asymptomatic or mild COVID-19 might have been not recorded. A 2–9% rate of breakthrough SARS-Cov-2 infections has been described after PrEP in lung and kidney transplant recipients during the omicron wave [11–14]. A shorter median follow-up after PrEP of 67 days was reported in these series [11]. In our study, a longer observation period and the linear trend in infections after PrEP may explain the higher rate (19%) of breakthrough infections. In the pooled retrospective studies, 10 out of 118 breakthrough infections after PrEP were severe which is comparable to the 6.4% in our study [11–14]. A pooled mortality of 2.7% in 112 COVID-19 breakthrough infections after PrEP has been published in kidney and lung transplant recipients from France and the U.S. in the era with dominant BA.1 and BA.2 variants [11–14].

Also, we could confirm the significant increase in SARS-CoV-2-S antibodies after PrEP in our study. Interestingly, there was no significant difference between single-dosed and double-dosed PrEP in our study.

The efficacy to prevent severe or critical disease by PrEP was demonstrated in a randomized controlled trial with 5197 participants [10]. There were notable differences aside from the much larger randomized trial, which might explain the different results in our study. First, the randomized trial was performed during the pre-delta era of the pandemic when severe and critical courses and death were more likely to occur. Second, just 3.3% of participants in the randomized trial were immunosuppressed in comparison to 100% in our study. Third, patients in the participants in the randomized trial were not vaccinated at inclusion while 90% in our study were fully vaccinated before PrEP. Fourth, our study was performed with a much higher incidence of SARS-CoV-2 infection during the study period.

In a retrospective analysis during the omicron era including 218 SARS-CoV-2 infected LTx patients from 8 German centers, 79% were treated with early antiviral therapy [20]. In our present study, 33% of patients did not receive a specific antiviral treatment. In patients with PrEP, almost half of the patients did not receive specific antiviral treatment and less frequently a combination therapy was applied. In general, transplanted patients are underrepresented in randomized clinical trials or antivirals against SARS-CoV-2. There are conflicting results about the efficacy in preventing severe disease or death in transplant patients by antiviral drugs [20–22] with considerable interaction potential in some agents [23].

Passive immunization by monoclonal antibodies is still an attractive concept for prophylaxis and early treatment of COVID-19 after organ transplantation because of a single-shot treatment with low toxicity and interaction potential as shown in our analysis. New formulations of monoclonal antibodies with enhanced activity against omicron variants are investigated as PrEP in clinical studies currently [24].

In conclusion, our study indicates that PrEP with tixagevimab and cilgavimab reduces breakthrough SARS-CoV-2 infections in lung transplant recipients. Furthermore, among those with an unfavorable risk profile, indicated by older age, comorbidities and low vaccination response, PrEP may have a favorable effect on the course of COVID-19. The study further demonstrates the decline of severe COVID-19 and associated death in a group of high-risk patients during the pandemic. The concept of passive immunization should be pursued and its success will be dependent on the development of monoclonal antibodies with efficacy against new virus variants.

### Supplementary Information

Below is the link to the electronic supplementary material.

Supplementary file1 (PDF 710 KB)

## Data Availability

Data are available on reasonable request from the corresponding author.

## Abbreviations

BAU: Binding antibody units
COVID-19: Coronavirus disease 2019
CLAD: Chronic lung allograft dysfunction
GFR: Glomerular filtration rate
LTx: Lung transplantation
PCR: Polymerase chain reaction
PrEP: Pre-exposure prophylaxis
SARS CoV2: Severe acute respiratory syndrome coronavirus type 2
SpO_2_: Blood oxygen saturation
