# Supplementary figures and images for: Efficacy of pre-exposure prophylaxis to prevent SARS-CoV-2 infection after lung transplantation: a two center cohort study during the omicron era
Source: Infection. 2023 Mar 16;51(5):1481–9. doi: 10.1007/s15010-023-02018-7 (PMC10018612; doi:10.1007/s15010-023-02018-7)

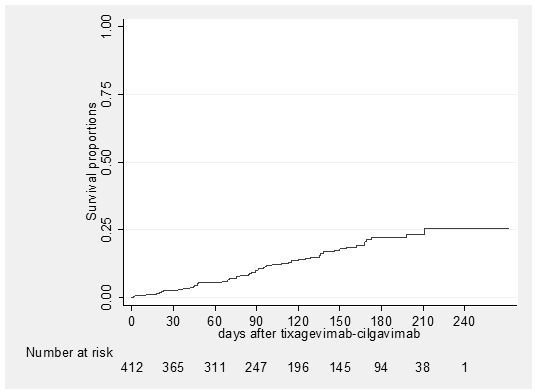

Supplement: Supplementary file 1 — Supplementary file1 (PDF 710 KB) [file 15010_2023_2018_MOESM1_ESM.jpg]
